# The role of Hoffa fat pad in total knee arthroplasty: A retrospective comparative study of functional and radiological outcomes

**DOI:** 10.1097/MD.0000000000046112

**Published:** 2025-11-21

**Authors:** Mahircan Demir, Hunkar Cagdas Bayrak, Ibrahim Faruk Adiguzel, Jan Zabrzyński, Selim Harmansa

**Affiliations:** a Department of Orthopaedics and Traumatology, Mamak State Hospital, Ankara, Türkiye; b Department of Orthopaedics and Traumatology, Cekirge State Hospital, Bursa, Türkiye; c Department of Orthopaedics and Traumatology, Etlik City Hospital, Ankara, Türkiye; d Department of Orthopaedics and Traumatology, Faculty of Medicine, Collegium Medicum in Bydgoszcz, Bydgoszcz, Poland; e Department of Orthopaedics and Traumatology, Eskisehir Yunus Emre City Hospital, Eskisehir, Türkiye.

**Keywords:** Hoffa, infrapatellar fat pad, pain, patellar tendon length, total knee arthroplasty

## Abstract

The infrapatellar fat pad (IPFP), or Hoffa fat pad, is frequently resected during total knee arthroplasty (TKA) to improve surgical exposure. However, its removal may affect patellar tendon integrity, vascular supply, and long-term functional outcomes. The role of the IPFP in osteoarthritis and its contribution to anterior knee pain remain topics of debate. To compare radiological and functional outcomes of patients undergoing TKA with either preservation or complete resection of the IPFP. This retrospective, observational study was conducted at a single institution between 2018 and 2021. Patients undergoing primary TKA for idiopathic osteoarthritis were divided into 2 groups according to the operating surgeon’s routine practice: IPFP preserved or IPFP resected. The primary outcomes were Insall–Salvati ratio, Oxford Knee Score, and Visual Analog Scale for anterior knee pain. Radiological and clinical evaluations were performed at 6 and 12 weeks, and annually up to 3 years postoperatively. At final follow-up, baseline demographics were comparable between groups. Postoperative Insall–Salvati ratio was 1.077 ± 0.12 in the excision group and 1.076 ± 0.12 in the preservation group (*P* = .93). Knee flexion improved similarly (115.8 ± 10.2° vs 116.0 ± 10.5°, *P* = .86). At 3 years, mean Oxford Knee Score was 20.3 ± 5.1 in the excision group and 19.9 ± 5.4 in the preservation group (*P* = .45), while mean Visual Analog Scale for anterior knee pain was 3.27 ± 0.4 versus 3.17 ± 0.4 (*P* = .064). Surgical time was shorter in the excision group (88 ± 8 vs 90 ± 6 minutes, *P* = .022); however, this small difference is unlikely to be clinically meaningful. Both groups demonstrated significant improvement in functional outcomes from baseline, with no long-term superiority of either approach. IPFP management during TKA does not significantly influence mid-term radiological or functional outcomes. Preservation may help maintain tendon integrity, whereas excision may modestly reduce operative time without affecting long-term patient-reported results. The choice to resect or preserve should therefore be based on intraoperative exposure and surgeon preference rather than expectations of improved outcomes.

## 1. Introduction

The infrapatellar fat pad (IPFP), also known as Hoffa fat pad (HFP), is often partially or completely excised during total knee arthroplasty (TKA) to improve the surgeon’s view of the tibial plateau and to reduce the risk of soft tissue interposition during prosthesis implantation.^[1]^ HFP consists of fibrous adipose tissue and is situated in the anterior compartment of the knee joint, an intracapsular but extrasynovial structure.^[2]^ It is bordered by the inferior pole of the patella, the patellar tendon, tibial plateau, and femoral condyles.^[3]^ The IPFP is a metabolically active tissue that secretes several cytokines and interleukins that may adversely affect the articular cartilage. It also contains nociceptive nerve fibers that secrete substance P, which can induce inflammatory mediators and contribute to anterior knee pain in osteoarthritis (OA).^[4]^ While its precise function is not fully understood, studies have hypothesized that it may contribute to knee biomechanics, promote lubrication, and serve as a reservoir for reparative cells after injury.^[5]^ However, due to its potential role in OA, the IPFP may also be considered an active osteoarthritic joint tissue.^[6]^

The removal of the IPFP during TKA using the traditional medial parapatellar approach remains controversial.^[7]^ A cadaveric study indicated that complete resection of the IPFP resulted in reduced contact pressures, less external tibial rotation, and medialization of the patella.^[8]^ Several investigations have examined patellar tendon length (PTL) after TKA, reporting that IPFP excision may lead to tendon shortening, increased anterior knee pain, and lower functional knee scores in short- to mid-term follow-ups.^[9]^ Conversely, other studies have demonstrated that IPFP resection does not significantly affect postoperative functional outcomes, pain scores, tendon morphology, or sonographic structure.^[10–12]^

More recently, several higher-quality studies have sought to clarify this controversy. Van Beeck et al reported in a systematic review that IPFP excision did not consistently impair outcomes but emphasized variability in study design.^[12]^ White et al similarly concluded that the effect of resection on pain and function was inconclusive due to heterogeneity.^[13]^ Sun et al, in a meta-analysis of randomized controlled trials, suggested that excision may increase the risk of anterior knee pain and patellar tendon shortening, although functional results were not significantly different.^[14]^ In contrast, Walker et al showed in a systematic review and meta-analysis that short-term outcomes were not superior with excision, and Benner et al, in a double-blind randomized clinical trial, found that fat pad resection did not affect postoperative function or gait.^[11,15]^

Despite these concerns, surgeons often excise the IPFP to facilitate tibial preparation, improve exposure, and minimize technical difficulties with bone cuts, cementing, and liner positioning. However, complete excision may compromise the vascular supply of the patellar tendon by damaging the lateral genicular artery, potentially leading to tendon scarring, stiffness, and patella baja, complications reported in association with TKA.^[16]^ Given the conflicting evidence and methodological limitations of prior studies, the present study was designed to investigate potential differences in the Insall–Salvati ratio (ISR) and Oxford Knee Score (OKS) between patients undergoing TKA with fat pad preservation and those with complete excision. The aim was to clarify the impact of IPFP management on postoperative functional and radiological outcomes.

## 2. Materials and methods

### 2.1. Study design

The study was approved by the local Bioethics Committee (Ethical Decision Number: ESH/BAEK 2024/21), and informed consent was obtained from all participants.

This was an observational, retrospective study conducted at a single institution between 2018 and 2021. Patients undergoing TKA for idiopathic OA were included. All procedures were performed by 2 experienced knee surgeons. Surgeon A consistently preserved the IPFP, whereas Surgeon B routinely resected it. No other differences existed in surgical approach or postoperative care between the 2 groups.

A posterior stabilized, cemented knee prosthesis was implanted in all cases using a midline medial parapatellar approach. The prostheses used were PMG (Tipmed, İzmir, Turkey) or Vanguard (Zimmer Biomet, Warsaw, Poland), fixed with OGM cement (OGM Group, Ankara, Turkey).

Inclusion criteria: primary TKA performed for idiopathic OA.

Exclusion criteria: secondary arthritis (e.g., rheumatoid arthritis [RA]), prior surgical procedures on the affected knee, severe deformity (>15° valgus or > 20° varus), advanced OA in other major joints, diabetes mellitus, severe peripheral arterial disease, and cases requiring augmentation with metaphyseal sleeves or bone substitutes due to major bone defects.

### 2.2. Surgical technique

All procedures were performed using an anteromedial approach with a tourniquet applied in each case. Antibiotic prophylaxis consisted of 2 g intravenous cefazolin.

**Figure 1.**
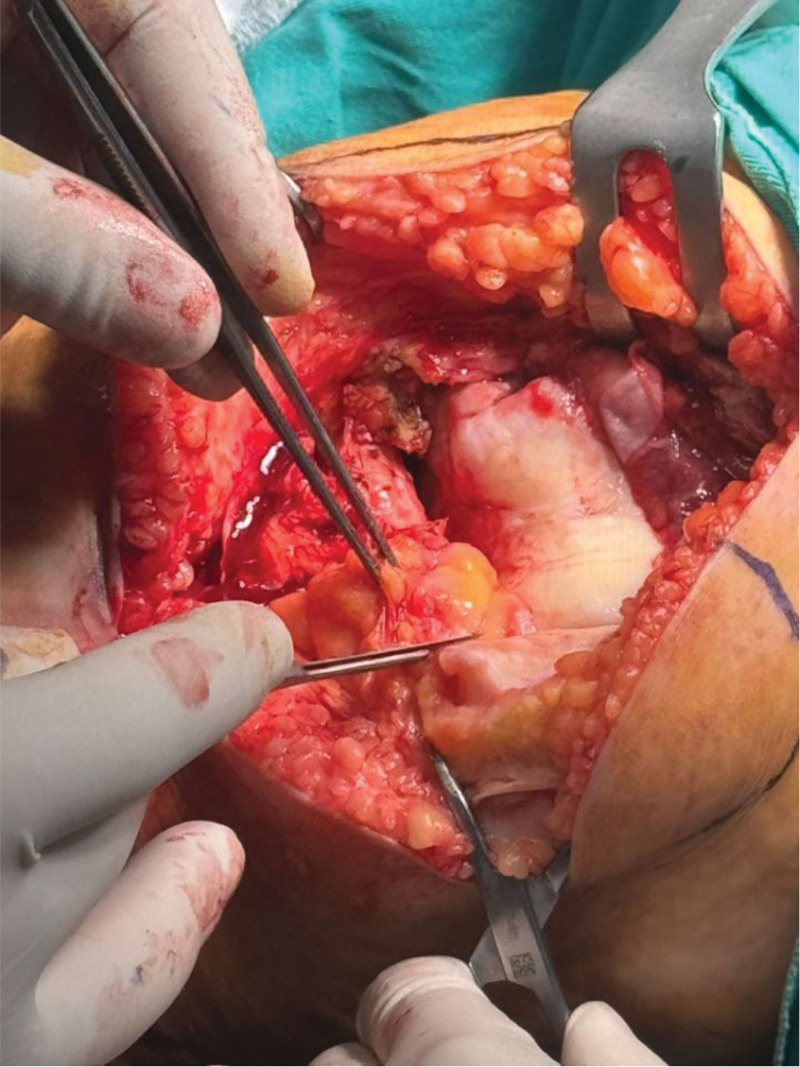
The moment of infrapatellar fat pad (IPFP) resection during total knee arthroplasty (TKA).

**Figure 2.**
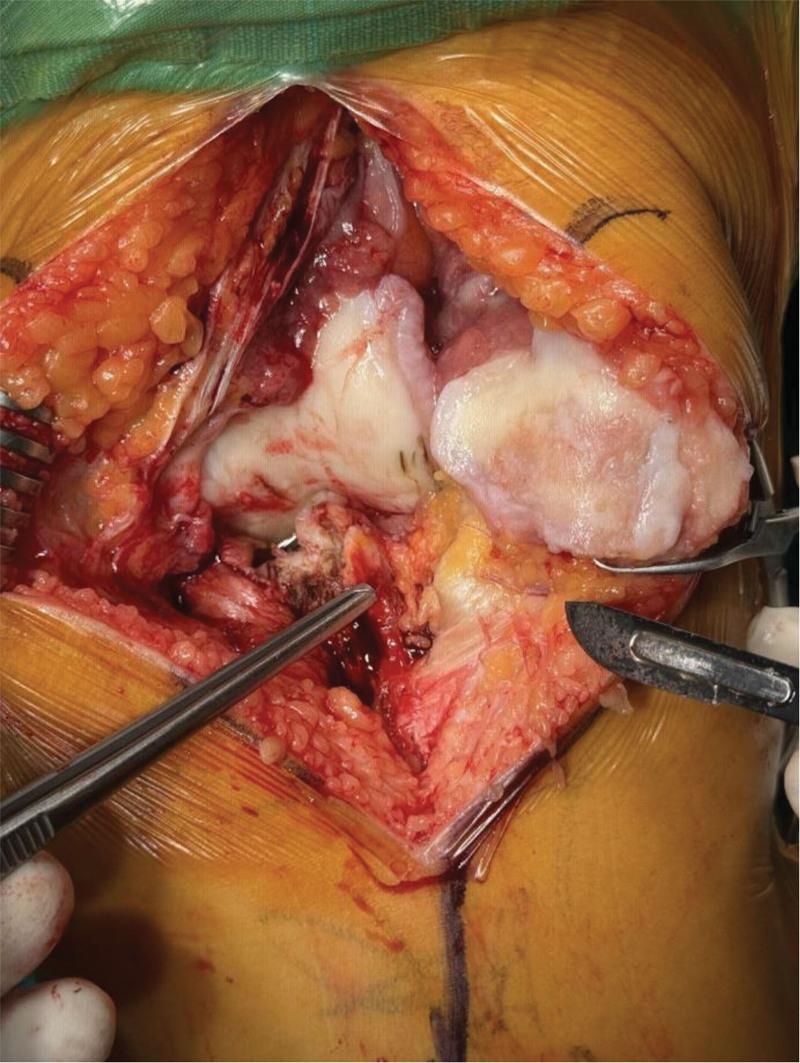
Infrapatellar fat pad (IPFP) after resection during total knee arthroplasty (TKA).

In the fat pad-preserving group, the IPFP was retained but incised along the medial border of the patellar ligament to improve exposure. In the fat pad-resection group, the IPFP was bluntly dissected from the patellar ligament and excised with a surgical blade (Fig. 1). In all cases, the anteromedial capsule was released from the tibia, but any residual IPFP attachments were left intact (Fig. 2).

All procedures followed the concept of mechanical alignment. Femoral components were implanted using a posterior referencing technique, and tibial rotation was set parallel to a line drawn from the posterior cruciate ligament.

Tranexamic acid was administered intravenously (1 g preoperatively and 2 g locally during surgery). A drain was placed in all patients and removed on the 1st postoperative day. Patients were mobilized after drain removal, with early ambulation and range of motion (ROM) exercises initiated the same day. Standard thromboprophylaxis consisted of 0.4 mL enoxaparin sodium subcutaneously once daily for 14 days.

### 2.3. Outcomes evaluation

Patients were assessed at 6 and 12 weeks, annually, and at 3 years postoperatively.

Radiographic evaluation: standard anteroposterior and lateral radiographs in 30° flexion were obtained. The ISR was measured to assess PTL. Radiographs were digitized and analyzed using PACS software (PiViewStar, Infinite Technology, Seoul, Korea). All measurements were performed with digital goniometers and precision tools with accuracy up to 1/1000 to minimize magnification and measurement errors.

Clinical and functional evaluation: functional outcomes were assessed with the OKS (In this version, higher scores indicate worse symptoms and poorer knee function). Anterior knee pain was evaluated using a Visual Analog Scale (VAS). Anterior knee pain was defined as any discomfort localized to the anterior knee during rest, stair climbing, or descending stairs.

Intraoperative parameters: surgical field visibility and operative time were noted.

Complications: infection, thromboembolic events, stiffness, and revision procedures were recorded.

### 2.4. Statistical analysis

Statistical analyses were conducted using IBM SPSS Statistics version 25 (IBM Corp., Armonk). Normality was tested using the Kolmogorov–Smirnov test. For parametric variables, between-group comparisons were made with the independent samples *t* test, and within-group comparisons with the paired samples *t* test. For nonparametric variables, the Mann–Whitney *U* test and Wilcoxon signed-rank test were used. Categorical variables were compared with the chi-square test. A *P* value < .05 was considered statistically significant. No adjustments were performed for multiple pairwise comparisons; therefore, *P*-values should be interpreted as exploratory.

## 3. Results

Of the 170 patients in the Hoffa excision group, 119 were women and 51 were men, whereas in the preservation group (n = 166), 108 were women and 58 were men. The mean body mass index was 29.9 ± 5.3 kg/m² in the excision group and 30.8 ± 4.2 kg/m² in the preservation group. Baseline demographic characteristics, including age, sex distribution, and body mass index, were comparable between the 2 groups (Table 1). No patients were lost to follow-up.

**Table 1:** Cohort characteristics.

	Hoffa excised n = 170	Hoffa protected n = 166	*P* value
Age	67.3 ± 4.2	69.7 ± 5.3	.05
BMI	29.9 ± 5.3	30.8 ± 4.2	.33
Gender	119 female/51 men	108 female/58 men	.334
Hgb decrease	1.61 ± 0.55	1.53 ± 0.55	.187
Surgery time	88 ± 8	90 ± 6	.022

BMI = body mass index, Hgb = hemoglobin.

The mean early postoperative ISR was 1.077 ± 0.12 in the excision group and 1.076 ± 0.12 in the preservation group (*P* = .932), showing no significant difference. Knee flexion improved similarly in both groups: preoperative mean flexion was 114.5 ± 11.9° in the excision group and 115.0 ± 11.4° in the preservation group, while at final follow-up it was 115.8 ± 10.2° and 116.0 ± 10.5°, respectively (*P* = .86).(Table 2).

**Table 2:** Functional outcomes.

Parameter	Preoperative Hoffa excised	Preoperative Hoffa protected	*P* value (preoperative)	Postoperative Hoffa excised 3rd year	Postoperative Hoffa protected 3rd year	*P* value postoperative	*P* value (preoperative vs postoperative
Oxford score	44.5 ± 6.4	43.5 ± 5.1	.054	20.3 ± 5.06	19.9 ± 5.4	.45	.001
Vas score	6.71 ± 0.6	6.58 ± 0.5	.078	3.27 ± 0.4	3.17 ± 0.4	.064	.001
Insall–Salvati ratio	1076 ± 0.12	1075 ± 0.12	.956	1077 ± 0.12	1076 ± 0.12	.932	.063/.131
Flexion angle	114.5 ± 11.9	115 ± 11.4	.78	115.8 ± 10.2	116 ± 10.5	.86	.007/.009

VAS = Visual Analogue Scale.

Functional outcomes demonstrated significant improvement in all patients. At 3 years, the mean OKS increased in both groups (excision group: 20.3 ± 5.06 vs preservation group: 19.9 ± 5.4, *P* = .45). The mean VAS for anterior knee pain at final follow-up was also comparable (3.24 ± 0.4 vs 3.17 ± 0.4, *P* = .064).

Surgical time was shorter in the Hoffa excision group (88 ± 8 min) compared with the preservation group (90 ± 6 min, *P* = .022). However, despite achieving statistical significance, the absolute difference of approximately 5 minutes is unlikely to be of major clinical relevance (Table 1).

Overall, both clinical and radiological outcomes improved significantly from baseline in all patients, and neither group demonstrated superiority in terms of long-term function or structural parameters (Table 2).

## 4. Discussion

In this retrospective comparative study, we found no significant differences between the HFP excision and preservation groups with respect to joint ROM, OKS, PTL, or anterior knee pain. These findings suggest that fat pad management (whether excision or preservation) does not substantially alter mid-term functional or radiological outcomes after TKA. Although surgical time was statistically shorter in the excision group (approximately 5 minutes), the absolute difference is unlikely to be clinically relevant. This nuance is important, as overemphasis on statistical significance without considering the clinical context could lead to misleading conclusions.

Our results align with several high-quality studies in the literature. Benner et al demonstrated in a double-blind randomized controlled trial that fat pad resection did not impair gait or function postoperatively.^[11]^ Van Beeck et al and White et al^[12,13]^ both concluded in systematic reviews that the available evidence does not consistently support harm or benefit from excision, although methodological limitations reduced the strength of their conclusions. Sun et al,^[14]^ in a meta-analysis of randomized controlled trials, suggested that excision may increase the risk of anterior knee pain and reduce PTL. Interestingly, our cohort did not reveal these associations, supporting the interpretation that differences observed in some meta-analyses may be related to study heterogeneity rather than a consistent biological effect. More recently, Walker et al^[15]^ emphasized that short-term outcomes were not superior following excision, reinforcing the view that fat pad removal provides no clear advantage.

Surgical exposure is often cited as the main justification for fat pad excision. The IPFP may obstruct visualization of the tibial plateau, and its removal can facilitate prosthesis placement and cementing.^[16]^ However, preservation does not appear to compromise surgical outcomes or increase complication rates. Indeed, our findings indicate that preservation can be performed safely without detriment to postoperative function. Furthermore, cadaveric and imaging studies have raised concerns about compromised patellar blood supply and tendon shortening following excision.^[17]^ Yet, clinical studies frequently fail to demonstrate a measurable difference, highlighting the redundancy and adaptability of the knee’s vascular network.

The IPFP is also biologically active, secreting inflammatory mediators, and neuropeptides such as substance P, which may exacerbate OA progression.^[18,19]^ This has led some investigators to hypothesize that excision could mitigate inflammatory cascades and improve outcomes. However, our results do not support a clinical benefit from routine resection in OA patients. Notably, outcomes may differ in other patient populations. A 2013 systematic review suggested that while OA patients may tolerate excision without significant detriment, RA patients experienced more discomfort and functional decline.^[20–22]^ Because our study included only OA patients, extrapolation to RA or other conditions must be made with caution.

Other systematic reviews have also highlighted conflicting results regarding pain, tendon length, and ROM.^[23–25]^ Some found moderate evidence of increased anterior knee pain following excision, whereas others observed no clear differences. This variability likely reflects methodological heterogeneity, differences in surgical technique, and variations in follow-up duration. Our prospective design and complete follow-up strengthen our findings by reducing attrition bias, yet residual confounding cannot be excluded.

The neurological role of the fat pad is another consideration. The IPFP is richly innervated with sensory and sympathetic nerve fibers that modulate pain and inflammation.^[26–28]^ Excision could theoretically alter this balance, contributing either to pain relief or increased sensitivity.^[29]^ While our study did not investigate histological or neurochemical changes, the absence of significant differences in VAS scores between groups suggests that any such effects were clinically negligible. Future translational studies correlating structural, molecular, and clinical outcomes could provide valuable insights into this question.

## 5. Limitations

This study has several limitations. First, no multivariable modeling was performed, limiting our ability to adjust for confounding factors such as comorbidities, baseline functional status, or subtle surgeon-related variables. Second, although both surgeons used standardized protocols, each consistently applied a single approach (excision vs preservation), which may introduce surgeon-related bias. Third, while follow-up was complete, the relatively modest sample size reduces statistical power for detecting small differences. Fourth, radiological measures of PTL were assessed retrospectively and may be subject to measurement variability despite standardized PACS-based methodology. Finally, we only investigated scalpel excision; the potential impact of electrocautery on bleeding, tendon preservation, or fibrosis remains an area for future research.

## 6. Conclusion

In conclusion, IPFP excision or preservation during TKA did not significantly influence postoperative functional or radiological outcomes in our cohort of OA patients. Although excision was associated with a statistically shorter operative time, the small difference is unlikely to be clinically meaningful. Taken together with prior randomized and systematic evidence, our results suggest that fat pad management should be individualized and based primarily on intraoperative exposure and surgeon preference, rather than expectations of long-term benefit. Future randomized controlled trials incorporating multivariable analysis and stratification by patient population (e.g., OA vs RA) are needed to further clarify the optimal approach.

## Author contributions

**Data curation:** Mahircan Demir.

**Formal analysis:** Hunkar Cagdas Bayrak.

**Investigation:** Mahircan Demir, Ibrahim Faruk Adiguzel, Selim Harmansa.

**Methodology:** Jan Zabrzyński.

**Writing – original draft:** Mahircan Demir.

## References

[1] Maculé F Sastre S Lasurt S Sala P Segur J Mallofré C. Hoffa’s fat pad resection in total knee arthroplasty. Acta Orthop Belg. 2005;71:714–7. 16459863

[2] Saddik D McNally EG Richardson M. MRI of Hoffa’s fat pad. Skeletal Radiol. 2004;33:433–44. 15221217 10.1007/s00256-003-0724-z

[3] Codorean II Codorean IB. Patella. In Clinical-MRI Correlations of Anterior Knee Pain: Common and Uncommon Causes. Springer Nature Switzerland; 2023;93168.

[4] Clockaerts S Bastiaansen-Jenniskens YM Runhaar J. The infrapatellar fat pad should be considered as an active osteoarthritic joint tissue: a narrative review. Osteoarthritis Cartilage. 2010;18:876–82. 20417297 10.1016/j.joca.2010.03.014

[5] İmren Y Dedeoğlu SS Çakar M Çabuk H Bayraktar TO Gürbüz H. Infrapatellar fat pad excision during total knee arthroplasty did not alter the patellar tendon length: a 5-year follow-up study. J Knee Surg. 2017;30:479–83. 27685767 10.1055/s-0036-1593360

[6] Yue S Zhai G Zhao S. The biphasic role of the infrapatellar fat pad in osteoarthritis. Biomed Pharmacother. 2024;179:117364. 39226725 10.1016/j.biopha.2024.117364

[7] Seo JG Lee SA Moon YW Lee BH Ko YH Chang MJ. Infrapatellar fat pad preservation reduces wound complications after minimally invasive total knee arthroplasty. Arch Orthop Trauma Surg. 2015;135:1157–62. 25986683 10.1007/s00402-015-2233-7

[8] Rathore S Vadlamudi N Lvsnr Y Kumar AA Reddy IV Krishnaiah K. Fat pad excision in total knee arthroplasty does not affect functional outcome or anterior knee pain at 1 year follow-up. J Arthroscopy Joint Surg. 2018;5:29–32.

[9] Yao B Samuel LT Acuña AJ. Infrapatellar fat pad resection or preservation during total knee arthroplasty: a systematic review. J Knee Surg. 2021;34:415–21. 31505700 10.1055/s-0039-1696692

[10] Michalak S Łapaj L Witkowska-Łuczak A Chodor P Zabrzyński J Kruczyński J. Resection of the infrapatellar fat pad during total knee arthroplasty has no impact on postoperative function, pain, and sonographic appearance of the patellar tendon. J Clin Med. 2022;11:7339. 36555955 10.3390/jcm11247339 PMC9782688

[11] Benner JL Bloemheuvel EM van der List JP Kerkhoffs GM van der Weegen W. Hoffa’s fat pad resection during total knee arthroplasty does not affect functioning and gait: a double-blind randomized clinical trial. Arch Orthop Trauma Surg. 2024;144:3657–68. 39196403 10.1007/s00402-024-05503-2 PMC11417071

[12] Van Beeck A Clockaerts S Somville J Van Glabbeek F Bos PK Van der Hart C. Does infrapatellar fat pad resection in total knee arthroplasty impair clinical outcome? A systematic review. The Knee. 2013;20:226–31. 23566735 10.1016/j.knee.2013.01.005

[13] White L Wraighte PJ Goldberg JA. The effect of infrapatellar fat pad resection on outcomes post-total knee arthroplasty: a systematic review. Arch Orthop Trauma Surg. 2016;136:701–8. 27003924 10.1007/s00402-016-2440-x

[14] Sun C Li X Song W Zhao J Wang Y Li L. Infrapatellar fat pad resection or preservation during total knee arthroplasty: a meta-analysis of randomized controlled trials. J Orthop Surg Res. 2020;15:297. 32758250 10.1186/s13018-020-01823-2 PMC7409474

[15] Walker H Rao A Tsimiklis J Smitham P. Are short term outcomes superior following total knee arthroplasty when infrapatellar fat pad is resected? A systematic review and meta-analysis. ANZ J Surg. 2024;94:1234–9. 38982806 10.1111/ans.19148

[16] Saxena S Patel DD Shah A Doctor M. Fat chance for hidden lesions: pictorial review of hoffa’s fat pad lesions. Ind J Radiol Imag. 2021;31:961–74. 10.1055/s-0041-1739383 PMC8817800 35136510

[17] Sellars H Yewlett A Trickett R Forster M Ghandour A. Should we resect Hoffa’s fat pad during total knee replacement? J Knee Surg. 2017;30:894–7. 28235234 10.1055/s-0037-1598039

[18] Michos J Karachalios T. Long term clinical outcome of total knee arthroplasty. The effect of limp alignment, implant placement and stability as controlled by surgical technique. In Total Knee Arthroplasty: Long Term Outcomes. Springer London; 2015;85–100.

[19] Salari P Baldini A. Revision knee surgery: the practical approach. EFORT Open Rev . 2021;6:495–500. 34267939 10.1302/2058-5241.6.210018 PMC8246103

[20] Mattap SMB. Utilising advanced imaging to understand joint pain and function limitations in older adults. Doctoral dissertation, University Of Tasmania; 2021.

[21] Nisar S Lamb JN Somashekar N Pandit H van Duren BH. Preservation vs. resection of the infrapatellar fat pad during total knee arthroplasty part II: a systematic review of published evidence. The Knee. 2019;26:422–6. 30738721 10.1016/j.knee.2019.01.007

[22] Naoum S. Should Hoffa’s fat pad be resected during total knee arthroplasty? A review of literature. Romanian J Military Med. 2022;125:196–201.

[23] Clancy Jr WG Narechania RG Rosenberg T Gmeiner JG Wisnefske DD Lange T. Anterior and posterior cruciate ligament reconstruction in rhesus monkeys. JBJS. 1981;63:1270–84. 7287797

[24] Haartmans MJ Emanuel KS Tuijthof GJ Heeren RM Emans PJ Cillero-Pastor B. Mass spectrometry-based biomarkers for knee osteoarthritis: a systematic review. Expert Rev Proteomics. 2021;18:693–706. 34228576 10.1080/14789450.2021.1952868

[25] Dragoo JL Johnson C McConnell J. Evaluation and treatment of disorders of the infrapatellar fat pad. Sports Med (Auckland, N.Z.). 2012;42:51–67. 10.2165/11595680-000000000-00000 22149697

[26] Ioan-Facsinay A Kloppenburg M. An emerging player in knee osteoarthritis: the infrapatellar fat pad. Arthr Res Ther. 2013;15:225–9. 24367915 10.1186/ar4422 PMC3979009

[27] Zeng N Yan ZP Chen XY Ni GX. Infrapatellar fat pad and knee osteoarthritis. Aging disease. 2020;11:1317–28. 33014539 10.14336/AD.2019.1116 PMC7505265

[28] Swift A. A clinical study exploring hip and knee osteoarthritis pain transmission using cerebrospinal fluid. Doctoral dissertation, University of Birmingham; 2012.

[29] Lehner B Koeck FX Capellino S Schubert TE Hofbauer R Straub RH. Preponderance of sensory versus sympathetic nerve fibers and increased cellularity in the infrapatellar fat pad in anterior knee pain patients after primary arthroplasty. J Orthop Res. 2008;26:342–50. 17902175 10.1002/jor.20498

